# Secure Enhancement for MQTT Protocol Using Distributed Machine Learning Framework

**DOI:** 10.3390/s24051638

**Published:** 2024-03-02

**Authors:** Nouf Saeed Alotaibi, Hassan I. Sayed Ahmed, Samah Osama M. Kamel, Ghada Farouk ElKabbany

**Affiliations:** 1 Department of Computer Science, College of Science and Humanities Al Dawadmi, Shaqra University, Dawadmi City 11911, Saudi Arabia; 2 Informatics Department, Electronics Research Institute, Cairo 12622, Egypt; hassanibrsayed@eri.sci.eg (H.I.S.A.); samah@eri.sci.eg (S.O.M.K.); gelkabbany@eri.sci.eg (G.F.E.)

**Keywords:** MQTT protocol, MQTT attacks, distributed machine learning, H2O distributed machine learning algorithms, security IoT

## Abstract

The Message Queuing Telemetry Transport (MQTT) protocol stands out as one of the foremost and widely recognized messaging protocols in the field. It is often used to transfer and manage data between devices and is extensively employed for applications ranging from smart homes and industrial automation to healthcare and transportation systems. However, it lacks built-in security features, thereby making it vulnerable to many types of attacks such as man-in-the-middle (MitM), buffer overflow, pre-shared key, brute force authentication, malformed data, distributed denial-of-service (DDoS) attacks, and MQTT publish flood attacks. Traditional methods for detecting MQTT attacks, such as deep neural networks (DNNs), k-nearest neighbor (KNN), linear discriminant analysis (LDA), and fuzzy logic, may exist. The increasing prevalence of device connectivity, sensor usage, and environmental scalability become the most challenging aspects that novel detection approaches need to address. This paper presents a new solution that leverages an H2O-based distributed machine learning (ML) framework to improve the security of the MQTT protocol in networks, particularly in IoT environments. The proposed approach leverages the strengths of the H2O algorithm and architecture to enable real-time monitoring and distributed detection and classification of anomalous behavior (deviations from expected activity patterns). By harnessing H2O’s algorithms, the identification and timely mitigation of potential security threats are achieved. Various H2O algorithms, including random forests, generalized linear models (GLMs), gradient boosting machine (GBM), XGBoost, and the deep learning (DL) algorithm, have been assessed to determine the most reliable algorithm in terms of detection performance. This study encompasses the development of the proposed algorithm, including implementation details and evaluation results. To assess the proposed model, various evaluation metrics such as mean squared error (MSE), root-mean-square error (RMSE), mean per class error (MCE), and log loss are employed. The results obtained indicate that the H2OXGBoost algorithm outperforms other H2O models in terms of accuracy. This research contributes to the advancement of secure IoT networks and offers a practical approach to enhancing the security of MQTT communication channels through distributed detection and classification techniques.

## 1. Introduction

Network and communication technologies have been developed to link the physical world to the digital realm through wireless communication. A large number of smart devices/objects can be connected without human interference. In 1999, Kevin Ashton proposed the concept of IoT, where many smart devices could be connected by linking radio-frequency identification (RFID) with the internet. It employs information and communication technology (ICT) to increase operational efficiency, share information, enhance the quality of people’s lives, and provide a luxurious lifestyle [1,2].

Smart objects in IoT can be connected using various methods, as defined by the Internet Architecture Board (IAB). IAB presented a set of guidelines for IoT smart object communication. There are four main communication models: device-to-device (D2D), device-to-cloud (D2C), device-to-gateway (D2G), and back-end data-sharing. 

Device-to-device (D2D), also referred to as machine-to-machine (M2M), is based on an IP network that allows two or more smart objects to communicate with each other and exchange data. To exchange data among smart objects, many protocols can be used to exchange messages such as ZigBee, Z-Wave, Wi-Fi, and Bluetooth [3]. With D2D communication, security is simplified due to the one-to-one relationship between the devices. Additionally, it has lower complexity, which can reduce its size and cost.

In device-to-cloud (D2C), also referred to as device-to-server (D2S), a smart object connects directly to internet cloud services based on a TCP/IP network or Wi-Fi connections. It often utilizes wired Ethernet or Wi-Fi connections; cellular technology can also be used. From a security perspective, this model is more complex than D2D because it involves two different types of network access credentials. To exchange data, many IoT application layer protocols are used to support the D2C communication model, such as the Message Queuing Telemetry Transport (MQTT) protocol, Extensible Messaging and Presence Protocol (XMPP), and Constrained Application Protocol (CoAP) [3]. 

With regard to device-to-gateway (D2G), smart objects can access cloud services via a local network gateway that includes application software to provide security and data protocol transmission between smart objects and the cloud, i.e., gateway devices can bridge the interoperability gap between devices that communicate on different standards. This kind of communication model provides interoperability between smart objects using different protocols such as Zigbee, Bluetooth, and Z-Wave protocols. Moreover, D2G supports the integration between smart objects and a legacy device [3].

With respect to back-end data-sharing, which is also referred to as server-to-server (S2S), this extends the D2C communication model, allowing authorized third parties to access smart devices and sensor data. This model supports the idea of exporting and analyzing data from a heterogeneous environment and sending it to an authorized user. The S2S model provides an integrated cloud application that facilitates the interoperability of smart objects in the cloud. In this approach, there are many protocols to allow various applications to exchange data, like Hypertext Transport Protocol (HTTP), Advanced Message Queuing Protocol (AQMP), and MQTT [3].

The structure of the IoT-based smart city contains six layers as follows: coding, perception, network, transport, application, and business. To manage the process in each layer, there are many IoT protocols such as connectivity protocols, communication protocols, data transmission/communication protocols, device management protocols, and security protocols. One of the most important protocols in IoT is the data transmission/communication protocol because it is used to communicate among smart devices. Several data transmission protocols exchange data between different devices, providing diverse features such as reliability, quality of service (QoS), performance, functionality, and scalability [4,5]. Many protocols are used to support the D2C, such as the Message Queuing Telemetry Transport (MQTT) protocol, Constrained Application Protocol (CoAP), Extensible Messaging and Presence Protocol (XMPP), and Advanced Message Queuing Protocol (AMQP) [4,5,6,7,8,9]. *MQTT* is a lightweight messaging protocol developed to facilitate efficient communication among smart devices. It supports devices to publish messages to specific topics, and other devices subscribe to those topics to receive the messages. MQTT is renowned for its low bandwidth requirements and low power consumption, making it ideal for resource-constrained devices and networks. MQTT is adopted for IoT applications because of its simple implementation model. Additionally, MQTT runs on the top of the Transmission Control Protocol (TCP) of the IoT network. *XMPP* enables real-time exchange of messages and presence data across devices over the internet. XMPP is extensible and supports the exchange of diverse data types, including text, images, and files. It finds wide application in various domains, such as chat applications, collaborative systems, and social networking platforms. *CoAP* is designed specifically for resource-constrained devices and low-power networks. It offers a lightweight and efficient means of communication between devices and web services. CoAP follows a client-server model and allows devices to interact with each other and access web resources using simple methods [4,5,6,7,8,9]. 

On the other hand, many protocols that permit various devices to exchange data are used to support the D2G. These protocols include:

1. Zigbee: A wireless communication protocol that offers to low-power, low-data-rate applications and is commonly utilized in home automation, industrial control, and sensor networks. Zigbee enables devices to form self-organizing networks with high reliability and extended coverage.
2. Bluetooth: A wireless communication technology widely employed for short-range data transmission between devices. It supports both point-to-point and point-to-multipoint connections. Bluetooth is commonly used to establish connections between devices like smartphones, tablets, headphones, and speakers. It facilitates data transfer, audio streaming, and device control, making it well-suited for applications such as personal area networks and smart home devices.
3. Z-Wave: A wireless communication protocol specifically designed for home automation applications. It enables devices to communicate with each other and relay messages. Z-Wave boasts low power consumption, secure communication, and interoperability among different Z-Wave-certified devices, making it an ideal choice for establishing a smart home ecosystem [3,5].

In addition, for S2S communication, the commonly used transport protocols are (1) HTTP, which is a request-response protocol, where the client initiates an HTTP request message to the server and then the server collects the requested resources and sends them back to the client; and (2) Advanced Message Queuing Protocol (AMQP), which handles publishers and consumers. Publishers produce messages, while consumers retrieve and process the messages. These protocols facilitate data exchange among multiple devices [3,4,5,6].

The MQTT protocol is utilized for the transfer and management of data among smart devices. It is a lightweight protocol that consumes low power, bandwidth, overhead, and memory; however, it lacks inherent security features [7]. Consequently, it is susceptible to various types of attacks, including port scanning (OS), HTTP flooding, ACK flooding, man-in-the-middle attacks, sniffing, buffer overflow, replay attacks, pre-shared key attacks, brute force authentication, Slow DoS against Internet of Things Environments (SlowITe), malformed data, distributed denial-of-service (DDoS) attacks, and MQTT publish flood attacks. Among these, the most prevalent and perilous attack against the MQTT protocol is the DDoS attack. This particular attack aims to deplete network bandwidth, CPU cycles, or memory resources, rendering services inaccessible to legitimate users [10]. Machine learning (ML) and deep learning (DL) approaches can be employed to detect and mitigate these attacks. 

In spite of the high accuracy of conventional machine learning algorithms in classifying network traffic as normal or attack, these methods are not capable of real-time operation and lack scalability in IoT environments. 

Furthermore, the complex mathematical aspects involved can be discouraging for many users. Among the available options, H2O is a suitable choice due to its ability to apply ML models to big data environments more rapidly than traditional methods. To tackle this issue, H2O streamlines various repetitive tasks, enabling developers to concentrate on outcomes rather than grappling with complexities. H2O’s AutoML simplifies the implementation of ML by providing a user-friendly interface for training and utilizing ML models. H2O AutoML automates the complete process of training multiple models, optimizing their hyperparameters and providing explanations for their performance [11].

H2O can effectively identify and classify different types of attacks targeting the MQTT protocol. H2O is an exceptional open-source ML framework that combines the advantages of being in-memory, distributed, and scalable. It offers rapid and efficient predictive analytics for handling large data sets. One of H2O’s distinguishing features is its impressive fast-scoring capabilities, generating predictions in sub millisecond times. It provides seamless integration with prominent big data platforms including Hadoop and Spark, demonstrating its versatility in addressing various data processing requirements. H2O’s adaptability and performance are further highlighted by its successful deployment on supercomputers in various high-performance computing (HPC) environments. This showcases its capability to meet the demands of complex and computationally intensive tasks. H2O supports parallel processing and distributed training, allowing the user to train models on big data clusters or cloud platforms. H2O supports scalability, which is crucial for tackling complex problems and achieving high-performance machine learning [12]. 

Therefore, the objective of this work is to propose a highly accurate classifier and detector of network traffic that can overcome these limitations. In IoT, the MQTT protocol serves as a communication coordinator for devices. Therefore, the aim is to propose a distributed classification approach that accurately detects attacks posing threats to this protocol. This study contributes to the development of ML models using H2O algorithms to detect, mitigate, and prevent MQTT attacks. This research focuses on evaluating different H2O algorithms such as random forests, generalized linear models (GLMs), gradient boosting machine (GBM), XGBoost, and deep learning algorithms to identify the most reliable security approach in terms of detection performance. Furthermore, by analyzing the transmission and dynamics of infected devices, the models developed in this work provide insights into the key features of compromised devices, enabling effective prediction and control of attack strategies. To achieve the underlying objectives, a distributed framework based on H2O is proposed to distinguish and categorize attacks that occur within the MQTT network. This approach leverages the power of modern computing clusters to efficiently process large data sets and perform complex ML tasks, thereby offering scalable and efficient solutions for detecting and preventing MQTT attacks. 

The paper is organized as follows: Section 2 provides a detailed description of the Message Queuing Telemetry Transport (MQTT) protocol, and its associated attacks. In Section 3, the proposed H2O framework for detecting and classifying MQTT attacks is described. Section 4 presents the performance evaluation of the proposed design. Finally, Section 5 concludes the paper.

## 2. Message Queuing Telemetry Transport (MQTT) Protocol

The Message Queuing Telemetry Transport (MQTT) protocol is a standard application layer protocol for IoT ecosystems. MQTT is based on the publish–subscribe paradigm broker and subscribers, which connect to a broker at any time. [8,9,13]. There are some important elements in the publish–subscribe model. First, a client/smart device sends messages through the broker over the network. A publisher contains data and publishes a message on a topic or subscribes to a specific topic. These topics are the way that clients register incoming messages. Second, messages contain the information about a client that wants to exchange a set of CONTROL packets between smart objects. The most important control packet types are named CONNECT, CONNACK, PUBLISH, PUBACK, SUBSCRIBE, SUBACK, and DISCONNECT [13,14]. 

### 2.1. The Operation Strategy of the MQTT Protocol and Its Attacks

As described above, once the TCP session is established, a publisher sends a request message to connect with the broker. This message is referred to as “CONNECT”. The broker responds with an acknowledgment message, “CONNACK”, to the publisher. The broker can manage and monitor all these messages and forward them to subscribers. Subscribers can receive these messages at different times. Consequently, the broker can implement authorization, message filtering, and message distribution among clients. Additionally, it can handle thousands of clients simultaneously [13,14].

The primary advantages of the publish–subscribe model include space decoupling, time decoupling, and synchronization decoupling. In terms of space decoupling, publishers and subscribers do not need to maintain extensive information about each other, such as IP addresses and ports. Furthermore, publishers and subscribers are located behind the MQTT broker’s firewall. Regarding time decoupling, publishers and subscribers do not need to operate simultaneously. Combining these two advantages, the publish–subscribe model can create more modular, robust, and secure software modules. Regarding synchronization decoupling, when publishing or receiving events occur, there is no need to block endpoint operations. Consequently, the publish–subscribe model operates in real-time communication, leading to increased scalability and reliability [13,14,15].

MQTT provides the QoS feature with three levels of message delivery: QoS 0, QoS 1, and QoS 2. Regarding QoS 0, MQTT messages are transmitted once without the need for confirmation or acknowledgment mechanisms. In the QoS 1 mechanism, MQTT messages are delivered at least once, with acknowledgment mechanisms. Regarding QoS 2, it is used to ensure that messages are delivered exactly once, without message duplication. 

MQTT protocol exploits many attacks that exhaust the network bandwidth, CPU cycles, or memory on the network to make services unavailable for legitimate users. These types of attacks include port scanning (OS), HTTP flooding, ACK flooding, man-in-the-middle attacks, sniffing, buffer overflow, replay attacks, pre-shared key attacks, brute force authentication, Slow DoS against IoT Environments (SlowITe), malformed data, DDoS attacks, and MQTT publish flood attacks. Among these, the most prevalent and perilous attack against the MQTT protocol is the DDoS attack. This particular attack aims to deplete network bandwidth, CPU cycles, or memory resources, rendering services inaccessible to legitimate users [10,16,17,18]. Attackers attempt to exploit key network parameters, which are typically represented by a queuing system characterized by factors such as packet arrival rate, queue length, and processing time [4,5,6,13].

### 2.2. Detecting MQTT Attacks

Several known attacks, including denial-of-service (DoS), man-in-the-middle (MitM), unauthorized access, message tampering, replay attacks, and others, pose significant threats to the MQTT protocol. DoS attacks can flood the MQTT broker with numerous connection requests or publish messages, overwhelming its resources and rendering it unresponsive. This, in turn, hinders legitimate clients from establishing connections or effective communication [1,2,3,4,5,6].

Detecting MQTT attacks is a paramount concern within the research community. This subsection highlights recent studies that employ mathematical models, detection methods, and artificial intelligence (AI) approaches to identify MQTT attacks. Khan et al. presented a deep neural network (DNN) for detecting attacks in the MQTT protocol [17]. Their evaluation encompassed binary and multiclassification scenarios, employing a DNN and other ML algorithms, including naive Bayes (NB), random forest (RF), k-nearest neighbor (KNN), decision tree (DT), long short-term memory (LSTM), and gated recurrent. An important limitation of this model is its inability to detect new types of attacks.

In an MitM attack, an attacker intercepts the communication between an MQTT client and the broker, enabling eavesdropping or modification of exchanged messages. By interfering with messages, attackers can manipulate transmitted data or inject malicious commands. Additionally, in cases where MQTT brokers lack proper security measures, attackers may exploit weak authentication mechanisms or default credentials to gain unauthorized access. Once inside, they can manipulate or disrupt MQTT communication. Celik et al. conducted a study on the security vulnerabilities in IoT devices, specifically focusing on the MQTT protocol [18]. They highlighted the issue of man-in-the-middle (MitM) attacks and how they can lead to privacy abuses. The study presented a case study of a smart home system application that employed the MQTT protocol for device communication. The authors emphasized the significance of addressing security concerns in IoT devices and proposed attack detection mechanisms and security measures for MQTT-based systems. The paper discussed the three critical aspects of information security: confidentiality, integrity, and availability. In addition, the authors reviewed previous studies on securing MQTT and discussed the limitations and vulnerabilities of the protocol. They proposed several approaches to detect attacks, authenticate communication, and secure MQTT communication. The paper also discussed the results of the attacks and evaluated the security risks and measures for MQTT. However, the study had a limitation in that it focused mainly on theoretical discussions and a simulated case study. It lacked real-world implementation or empirical validation of the proposed security measures. 

Kurdi and Thayananthan [13] presented a multitier MQTT architecture with multiple brokers based on fog computing to secure the Industrial Internet of Things (IIoT). They addressed the challenges posed by the rapid growth of internet-connected devices and the limitations of existing authentication mechanisms. The authors introduced a lightweight mutual authentication scheme based on a hash function and XOR operation, which deployed authentication managers with each broker. Although this system was designed to resist cyberattacks and provide scalability, it lacks in-depth implementation details and comprehensive evaluation. Additionally, it does not include a discussion of the feasibility or challenges of deploying its architecture in real-world industrial settings [13]. 

### 2.3. Smart Home IoT Network

As the demand for large-scale IoT deployments continues to rise, the management of access and administration for numerous devices presents significant challenges in terms of network bandwidth, communication protocols, and platform service architecture. IoT protocols play a crucial role in addressing various communication concerns of smart devices, including navigating complex and unreliable network environments, accommodating limited memory and flash memory capacity, and working with constrained processing capabilities. To address these challenges, the MQTT protocol was developed. It offers several benefits, such as high efficiency, reliable messaging, extensive support for connections, and secure bidirectional communication. Nevertheless, there are notable security issues, including DoS attacks, identity spoofing, information disclosure, elevation of privileges, and data tampering [6,7]. 

**Figure 1:**
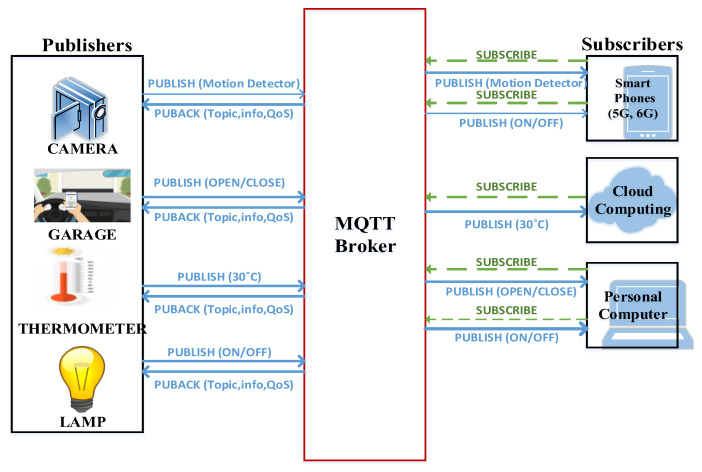
Smart home IoT network: Leveraging the MQTT protocol.

The smart home comprises various sensors, such as a camera, garage door sensor, thermometer, and lamp sensor, all communicating with a central broker. These sensors collect and transmit crucial information, including motion detection, garage door status (open/close), temperature measurements, and light status (ON/OFF). On the receiving end, there are three subscribers, which include smartphones and a personal computer, configured to receive updates from the four sensors, as depicted in Figure 1. For instance, the camera sensor publishes data from the motion detector to the broker only when it detects any movement. Similarly, the garage door sensor reports its status (open/close), and the lamp sensor communicates its status (on/off) to the broker. The thermometer sensor periodically publishes temperature measurements, which are also sent to the broker. The broker plays a vital role in managing and processing the data from these sensors. Consequently, subscribers, including smartphones, 5G/6G devices, cloud services, and personal computers, can subscribe to the data generated by all these sensors.

### 2.4. Phases of the MQTT Attacking System

The phases of the MQTT attacking system involve various techniques used by attackers to compromise the MQTT protocol and disrupt its normal operations. These attacks aim to manipulate MQTT messages and compromise the integrity of the publish–subscribe model [3,4,5,6]. The attacking system can be divided into three distinct phases:

1. Connection attack phase: In this initial phase, the malicious nodes target the connections within the MQTT network. They may attempt to intercept and tamper with MQTT messages, altering their content before forwarding them to the intended recipient. This can deceive the recipient or trigger unintended actions based on manipulated data.
2. Authentication attack phase: Following the connection phase, the attackers focus on undermining the authentication mechanisms in place. They may try to bypass authentication protocols or exploit vulnerabilities to gain unauthorized access to the MQTT network.
3. Communication attack phase: The final phase involves attempts to disrupt the communication channels within the MQTT network. Attackers may employ techniques such as replay attacks, where they capture MQTT messages and later retransmit them to the broker or intended recipient. This can result in action duplication or unauthorized access when sensitive information or commands are involved.

Phase one: The connection attack phase

**Figure 2:**
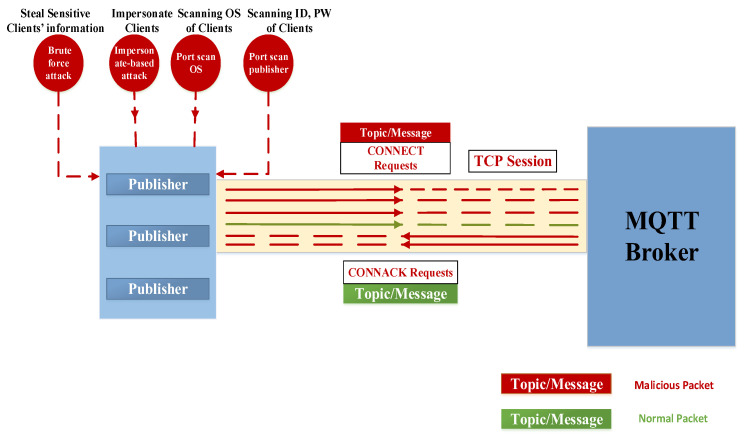
The connection attack phase.

As depicted in Figure 2, during this phase, the attacker’s objective is to acquire client information and employ victim/client credentials (username and password) to establish a connection with the broker. The attacker may initiate a flood of CONNECT requests directed at the broker, originating either from a single source or multiple sources. Consequently, multiple TCP sessions are initiated, and the broker responds by sending CONNACK packets to both legitimate and illegitimate clients [6,7].

Phase two: The authentication attack phase

**Figure 3:**
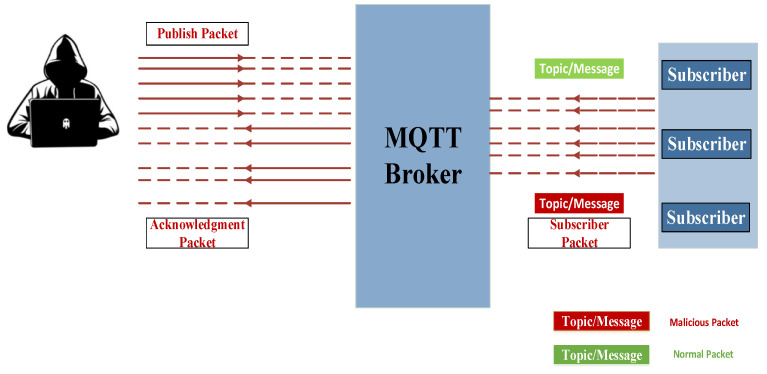
The authentication attack phase.

During this phase, once the TCP connection is established, the attacker proceeds to send a series of connection requests to the broker to gather sensitive information and compromise the authentication mechanism. Subsequently, the attacker launches multiple publishers and subscription control packet requests with invalid authentication credentials. This causes the broker to send numerous acknowledgment requests, rendering it consistently occupied. Furthermore, the attacker may manipulate the payload of subscription control packets by introducing multiple topics using wildcards. This manipulation enables the attacker to receive messages from all topics, regardless of whether they match or not. Consequently, this unauthorized access potentially grants the attacker entry into the network. Figure 3 provides a visual representation of the authentication attack, illustrating the steps involved and the potential consequences arising from the attacker’s actions.

Phase three: The communication attack

As a result of the flooding of the control packets, the time interval between subsequent PUBLISH packets increases. As such, the arrival rate (*λ*) will increase, the sleep-interval time will subsequently decrease, and the arrival rate of attacked requests will exceed the average processing time of the system: (ω). *λ* > ω. Consequently, an attacker can exhaust CPU capacity, and the broker may drop legitimate requests. 

**Figure 4:**
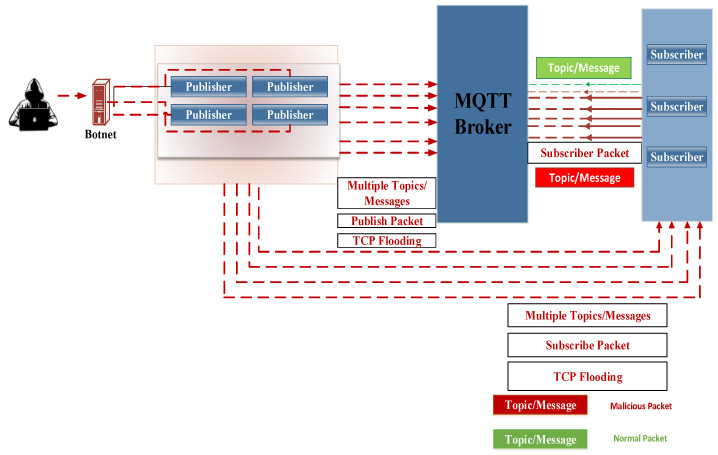
The communication attack phase.

In the communication attack phase, the attacker can send malformed connect packets to the broker, resulting in network damage. By manipulating elements such as the payload size, WILL messages, WILL topics, and WILL QoS, the attacker can increase the size of control packets. This increase in size leads to higher consumption of bandwidth resources, CPU resources, and processing time. Attackers can also manipulate the Duplicated Delivery of Publish Connect Packets (DUPs) by altering communication and control packet transfers. A zero DUP value compels the client to send a publish connect packet, while a DUP value of one force the client to resend previous packets multiple times, causing a flood of duplicated messages and undermining the concept of QoS2. Additionally, the attacking system may inundate the broker with a high volume of subscription requests combined with random topics in the same messages. This flooding tactic has a detrimental impact on system performance and disrupts the QoS levels. Figure 4 illustrates the communication attack phase, offering a visual representation of the attack scenarios and their potential consequences.

Researchers have made significant efforts to develop mechanisms for detecting MQTT attacks and enhancing security through AI, ML, and DL approaches. However, these methods may not always provide the necessary strength or accuracy for effective attack detection. Moreover, traditional ML methodologies may not be well-suited for big data environments, limiting their effectiveness in detecting and mitigating MQTT attacks. Further research and development are essential to create robust and reliable mechanisms for securing MQTT communications. 

The aim of the current study is to build a distributed processing framework for the MQTT protocol attack detection and mitigation. H2O offers a fully open source, distributed in-memory, fast linear scalable, and predictive analytics framework. One of its advantages is its ability to distribute data across the cluster and store it in a compressed columnar format, which allows users to read data in parallel. H2O supports the most widely used machine learning algorithms. The proposed framework exploits the characteristics of the distributed machine learning H2O for the MQTT protocol attack detection and mitigation. In this work, a security framework is deployed within a smart home IoT network that utilizes the MQTT application protocol. The next section discusses the proposed framework in detail. 

## 3. The Proposed Framework

This research addresses the urgent need to enhance the security of MQTT and contributes to the advancement of secure IoT networks. It provides a practical solution for securing MQTT communication channels by proposing a framework that utilizes distributed machine learning algorithms based on H2O. The framework aims to improve the security of the MQTT protocol in various networks, including the IoT, by leveraging H2O algorithms and architecture. It enables real-time monitoring and detection of abnormal behavior in MQTT communication channels, facilitating the identification and mitigation of potential security threats. Furthermore, the proposed framework conducts a comprehensive evaluation of different H2O algorithms to identify the most reliable ones for detecting MQTT attacks. 

To maintain the MQTT protocol from cyberattacks, a security system has been developed within the proposed framework. This system aims to detect and mitigate the effects of the attacks executed by the malicious nodes by understanding and addressing the vulnerabilities associated with the above phases (described in Section 2.3 in detail).

### Distributed and Secure Broker (DSBroker)

The distributed detector broker plays a crucial role in enhancing security. Attack detector agents are responsible for analyzing IoT network traffic in a distributed manner, utilizing multiple core processing units. Each instance of traffic is treated as an input data set and processed concurrently in real-time analysis.

Every sensor within the network receives a data set, treated as an instance, and forwards it to the nearest processing unit. These processing units are strategically located close to the sensors to increase the broker’s speed and enable faster response times. Additionally, these processing units handle all input data sets, encompassing various types of attacks and normal traffic. The primary objective of these processing units is to minimize the computational load associated with attack detection, thereby reducing the cost of the DSBroker (distributed and secure broker) detection agent.

**Figure 5:**
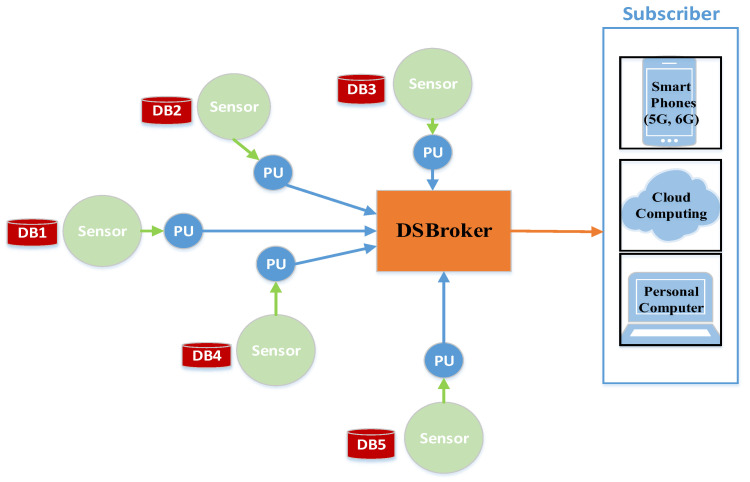
DSBroker (distributed and secure broker).

The DSBroker detection agent is responsible for managing the publish–subscribe model, preventing the duplication of messages, and distributing processing tasks to enhance the accuracy rate of the publish–subscribe model. As a result, it efficiently manages the processing time and elevates security accuracy. The DSBroker detection agent is also equipped to detect flow rates, monitor MQTT packet length and field length, and identify any unusual behavior occurring within specified time intervals. Figure 5 illustrates the DSBroker detection agent, offering a visual representation of its role within the framework.

## 4. Implementation and Results

To efficiently apply various ML algorithms to large data sets, the H2O framework is employed. H2O is a fully open-source, distributed, in-memory, and fast, linearly scalable predictive analytics framework. One of its notable advantages is its capability to distribute data across a cluster and store it in a compressed columnar format, enabling parallel data reading. H2O provides support for a wide range of commonly used ML algorithms, including GBM, GLM, DL, and more. Furthermore, H2O boasts an industry leading AutoML functionality that automatically explores different algorithms [19,20]. H2O AutoML is accessible through Python, R, Java, Scala, and a web-based graphical user interface. In this study, the H2O framework is harnessed to identify and classify attacks to which the MQTT protocol is susceptible. Subsequent subsections provide a concise overview of the MQTT data set, explore the features of H2O, outline the ML algorithms employed, and establish the evaluation metrics utilized to assess the performance of various ML algorithms.

### 4.1. Data Sets

This research is conducted using the MQTTset data set, which has been made available by Vaccari et al. [21]. The MQTTset data set comprises data collected from eight MQTT sensors, each possessing distinct features. Each sensor is associated with a specific data profile and topic, which are linked to the MQTT broker. The data profile specifies the type of data that the sensors transmit, while the topic is determined by the sensor when it sends data to the broker. The MQTTset data set has been designed for use within an IoT network based on the Message Queue Telemetry Transport (MQTT) protocol, a publish–subscribe protocol [21]. The MQTTset was generated utilizing the IoT-Flock network traffic generator, an open-source IoT tool designed for generating IoT network traffic. This traffic data set encompasses both normal and attack traffic, replicating real-time network conditions. IoT-Flock is equipped with the capability to configure network scenarios, including node configurations (such as device IP addresses device types, ports, etc.) and the communication interactions between the sensors and the broker. IoT-Flock operates with a three-layer structure, encompassing the presentation layer, business logic layer, and network layer. Within IoT-Flock, there exist four types of attacks: MQTT packet crafting attacks, MQTT publish flood, CoAP segmentation fault attack, and CoAP memory leak attack [22].

The MQTTset data set includes both legitimate and malicious traffic for IoT devices. It can simulate the IoT environments, such as smart homes and smart buildings. The MQTTset data set contains a total of 165,281 malicious traffic samples and 11,915,716 legitimate traffic samples. There are 130,223 packets related to flooding DoS, 613 packets for MQTT publish flood, 9202 packets for SlowITe, 10,924 packets for malformed data, and 14,501 packets for brute force authentication. The MQTTset data set comprises a total of 1,048,576 data samples, with 7,340,032 samples allocated for training purposes and 3,145,728 samples for testing purposes. Within the MQTTset data set, there are 33 distinct features that cover various aspects, including TCP flags, time delta, length, connection acknowledgment flags, as well as MQTT-specific attributes such as message type, QoS, and topic details.

### 4.2. The Key Features of the H2O Framework

The H2O framework distinguishes itself through its robust support for both supervised and unsupervised ML algorithms, distributed computing capabilities, and automated machine learning (AutoML) tools. ML models are trained using H2O AutoML, progressing from experimentation to production, without requiring an in-depth understanding of complex statistics or data science. H2O’s memory management combines in-memory and distributed processing techniques to efficiently handle vast volumes of data. This framework facilitates straightforward deployment through optimized Java and model objects. H2O offers compatibility with multiple programming languages and operating systems, ensuring a versatile and user-friendly environment. Additionally, its graphical user interface (GUI) seamlessly integrates with popular web browsers. The architectural design of the H2O software stack revolves around the interaction between REST API clients and components within the H2O JVM process (H2O_cluster_version is 3.40.0.4, H2O_cluster_memory is 7.461 Gb, localhost, and Python_version is 3.10.10). REST API clients communicate with H2O via socket connections. H2O has proven itself as a powerful tool for constructing highly accurate ML models, particularly when dealing with extensive data sets. Its AutoML tools simplify the training and tuning processes, making them accessible to both novice and experienced users.

The efficiency and scalability of the H2O framework, combined with its diverse array of ML algorithms, position it as a preferred choice for big data applications. The effectiveness of H2O’s AutoML functionality is underscored by benchmark comparisons, underscoring its role in facilitating collaborative work among diverse teams to achieve common objectives [23].

### 4.3. The Evaluation Metrics of the H2O

H2O strengthens widely used ML and statistical algorithms, encompassing deep learning, generalized linear algorithms, and gradient boosting machines. Its leading AutoML functionality automates the tuning of hyperparameters and the selection of algorithms to generate the best supermodel. This framework enjoys widespread popularity within the Java, R, and Python communities and boasts a global user base of over 18,000 consortiums. To utilize H2O effectively, the H2O package must first be installed. Subsequently, an instance of the desired algorithm with specified hyperparameters should be created. The H2O model is then trained using the provided data set, and its performance is evaluated on a validation data set. Finally, the trained algorithm can be used to make predictions on new data. H2O provides various built-in metrics for evaluating performance, including mean squared error (MSE), root-mean-square error (RMSE), mean per class error (MCE), and log loss [24]. These metrics facilitate the comparison of different models, aiding in the selection of the most suitable model for a specific application.

▪ Mean Squared Error (MSE): The average squared error between actual and predicted values. MSE is calculated as follows:

MSE = 1/n ∗ SUM[(y_i − ŷ_i)^2^] (1)

where “n” is the number of observations in the data set, “y_i” is the actual value of the target variable for the i-th observation, and “ŷ_i” is the predicted value of the target variable for the ith observation.

▪ Root-Mean-Square Error (RMSE): The standard deviation of the prediction errors. RMSE is calculated as follows:

RMSE = SQRT(MSE)(2)

▪ Log Loss: Logarithmic loss indicates how close a prediction probability is to the actual/corresponding real value. It is calculated as follows:

LogLoss = −1/n ∗ SUM[y_i ∗ LOG(p_i) + (1 − y_i) ∗ LOG(1 − p_i)](3)

where “p_i” is the predicted probability that the i-th observation belongs to the positive class, and “y_i” is the actual binary label of the i-th observation (0 or 1).

▪ Mean Per Class Error (MCE): The average of the errors of each class in a multiclass data set. MCE is defined as follows:

MCE = 1/K ∗ SUM[error_rate(c)](4)

where “K” is the number of classes, and error_rate(c) is the error rate (proportion of misclassified instances) for class for class c. The error rate for a given class c is calculated as follows:

error_rate(c) = FP(c) + FN(c)/TP(c) + FN(c) + TN(c) + FP(c)(5)

where “TP(c)” is the number of true positives for class c, “FN(c)” is the number of false negatives for class c, “TN(c)” is the number of true negatives for class c, and “FP(c)” is the number of false positives for class c.

### 4.4. Machine Learning Algorithms

Machine learning algorithms can be categorized into two main classes: supervised and unsupervised learning algorithms. Supervised algorithms, including random forests, GLM, GBM, XGBoost, and DL algorithms, rely on labeled data sets designed to train algorithms for accurate data classification and outcome prediction. These algorithms require predefined labels to learn and make predictions effectively. On the other hand, unsupervised models such as the generalized low-rank model (GLRM), k-means, and principal components analysis (PCA) operate on unlabeled data sets [25]. They are tasked with identifying hidden patterns and structures within data without the need for human intervention.

Choosing the appropriate approach depends on several factors, including the volume of the data and the specific problem definition. In cases where high accuracy and trustworthiness are required, especially for handling big data, supervised learning algorithms are a suitable choice. In this research, supervised learning algorithms, specifically H2O supervised models, are employed to detect and predict MQTT attacks. 

The subsequent subsection provides detailed information on the necessary steps and considerations for various H2O distributed ML and its implementation to identify the most reliable ones for detecting MQTT attacks. 

#### 4.4.1. Distributed H2O Random Forest (DRF)

Distributed random forest (DRF) is an ML algorithm that is particularly well-suited for detecting attacks in distributed environments. It excels at identifying patterns and anomalies in various data subsets, thereby enhancing the accuracy and robustness of the detection model. Furthermore, due to its distributed nature, DRF can efficiently handle large volumes of data without overwhelming individual machines or nodes. DRF operates by constructing a decision tree forest, where each tree is built using a random subset of the data and features. In a distributed environment, data is typically distributed across multiple machines or nodes. DRF leverages this architecture by creating decision trees on each node using its local data. These individual trees are then combined into a single “forest” of trees, with each tree’s output weighted according to its accuracy. When a new instance requires classification, the forest transmits or broadcasts it to each tree in parallel. The final classification result is obtained by aggregating the outputs of all the trees. One notable advantage of DRF is its ability to utilize nonlinear algorithms. Additionally, it can provide valuable feedback on the importance of each predictor in the algorithm, making it a robust choice for handling noisy data [26,27,28,29]. When employing DRF for MQTT attack detection, a series of five steps are typically followed. A detailed pseudocode description of these steps is as follows:

1. Data preprocessing:
  ▪ Read the input data from MQTT logs or other sources
  ▪ Prepare the data by encoding categorical variables, normalizing numerical values, and removing any irrelevant or redundant features
2. Train/test split:
  ▪ Split the preprocessed data into training and testing sets, e.g., using an 80/20 split
3. Distributed random forest model building:
  ▪ Partition the training data into “k” subsets
  ▪ For each subset “k”, select a random subset of features “F_k_”
  ▪ Build a decision tree “T_k_” on partition “D_k_ “with features “F_k_”
  ▪ Combine individual trees “T_k_” into a single forest of trees “F” with associated weights “w_k_” proportional to accuracy (T_k_, V)
  ▪ Return the forest “F” and associated weights “w_k_”
4. MQTT attack detection:
  ▪ When presented with new MQTT data “x”
  ▪ Pass it down to all trees in parallel and collect their outputs
  ▪ Aggregate the outputs by taking the majority vote:
    y_pred = mode(T_1_(x), T_2_(x), …, T_K_(x)) (6)
  ▪ If y_pred indicates an anomaly, raise an alert or take appropriate action
5. Model evaluation:
  ▪ Evaluate the performance of the distributed random forest algorithm on the test set using one or more metrics from the metrics described above (MSE, RMSE, or LogLoss)
  where:
  ▪ “D” is the input data set
  ▪ “K” is the number of nodes or machines used in the distributed environment
  ▪ “F_k_” is the subset of features used in node “k”
  ▪ “T_k_” is the decision tree built on partition k and features “F_k_”
  ▪ “w_k_” is the weight assigned to tree “T_k_” based on its accuracy on validation data set “V”
  ▪ T_1_(x), T_2_(x), …, T_K_(x) are the predictions of each tree on data sample “x”
  ▪ “y_pred” is the final prediction indicating whether the MQTT data “x” represents a normal or anomalous behavior.

The architectural details, training outcomes, and evaluation results of the DRF models are summarized in Table 1. 

#### 4.4.2. Distributed Gradient Boosting Machine (DGBM)

The distributed gradient boosting machine (DGBM) is an extension of the well-known gradient boosting estimator algorithm, tailored to manage massive data sets that surpass the processing capacity of a single machine. DGBM achieves this by distributing the computational load across multiple machines or nodes. A significant feature of DGBM is its ability to parallelize the training process, which is accomplished by partitioning the data set into smaller subsets that are distributed across these machines. Each machine independently executes a model on its designated subset of the data. At the conclusion of this process, the results from all models are combined to create a final ensemble model. Another noteworthy characteristic of DGBM is its proficiency in minimizing data transfer between machines. It employs communication-efficient algorithms, mitigating the communication overhead bottleneck that can occur in distributed systems.

Various implementations of DGBM exist, including XGBoost and LightGBM [30,31,32]. These libraries enjoy widespread usage in both industry and academia and have consistently delivered state-of-the-art performance across various ML tasks. In essence, DGBM stands as a potent ML algorithm, enabling the training of large-scale models on distributed systems. Its capability to parallelize training and employ communication-efficient algorithms renders it a preferred choice for numerous real-world applications.

▪ The objective function [33]:

L(y, F) = Σ l(yᵢ, F(xᵢ)) + Ω(F)(7)

where “y” is the vector of true labels for the training data, “f” is the sum of the outputs from all the decision trees, “xᵢ” is the feature vector for the i-th data point, and “l” is the loss function used to calculate the difference between the predicted output and the true label. Additionally, Ω(F) is the regularization term that controls the complexity of the model.

▪ The gradient and Hessian for the loss function [34]:

gᵢ = ∂ l(yᵢ, F(xᵢ))/∂ F(xᵢ)(8)

hᵢ = ∂^2^ l(yᵢ, F(xᵢ))/∂ F(xᵢ)^2^ (9)

where “gᵢ” is the gradient and “hᵢ” is the Hessian for the i-th data point.

▪ The prediction from the k-th decision tree can be calculated as follows:

f(x) = fk_−1_(x) + ρ·h(x) (10)

where “fk_−1_(x) ” is the prediction from the k − 1-th decision tree, “ρ” is the learning rate or step size, and h(x) is the average of the Hessians over all the data points.

▪ The weights or distribution over the data points can be calculated as follows [35]:

wᵢ = exp(−yᵢ·fk_−1_(xᵢ))/Σexp(−yᵢ·fk₋_1_(xᵢ)) (11)

where “wᵢ” is the weight assigned to the i-th data point and Σ is the sum over all the data points. Equations (8) and (9) are used iteratively to train the model on a distributed system where each node is responsible for a subset of the data. 

The following steps (the pseudocode) are used for MQTT attack detection when using the DGB machine:

1. Load the MQTT traffic data
2. Preprocess the data to extract relevant features such as packet length, message type, and topic name
3. Split the data set into training and testing sets
4. Initialize the DGB machine model with appropriate parameters
5. Train the model using the training data set
6. Evaluate the performance of the model (using the testing data set)
7. For each incoming MQTT message:
  - Extract the relevant features from the message
  - Use the trained model to predict whether the message is malicious or benign
  - Take appropriate action based on the prediction, such as blocking or flagging the message

As shown in Table 2 a summary of the system performance observed when employing DGBM models for MQTT attack detection is provided.

##### H2O Distributed XGBoost Model (H2OXGBoost)

Distributed XGBoost (DXGBoost) is a distributed implementation of the XGBoost algorithm [36], purposefully designed to handle large data sets efficiently. The XGBoost algorithm is a highly optimized gradient boosting library that implements ML algorithms within the framework of GBM. Notably, XGBoost is known for its versatility because it can be implemented using various programming languages, including R, Python, and Java. This flexibility makes both XGBoost and H2OXGBoost two of the most preferred GBM frameworks across a wide range of applications [37]. XGBoost, including its distributed counterpart DXGBoost, employs decision trees as base learners for gradient boosting. However, it incorporates a range of optimizations that enable it to scale seamlessly to data sets encompassing billions of rows and thousands of columns [38]. In addition to its scalability and adept handling of missing values, H2OXGBoost offers several additional features to streamline model training and tuning. These features include early stopping, which permits users to halt the training process prematurely if the validation error ceases to improve [39], and grid search, an automated approach to hyperparameter tuning that explores a predefined set of hyperparameters.

XGBoost and H2OXGBoost Model Description:

▪ The objective function of the XGBoost algorithm is given by [40]

obj = L + Ω(12)

where “L” is the loss function that measures the difference between the predicted and actual values, and “Ω” is the regularization term that penalizes complex models.

▪ The prediction of a single decision tree in XGBoost is given by

f(x) = wq(x)(13)

where “w” is the weight assigned to each leaf node, and “q(x)” is the indicator function that outputs “1” if “x” belongs to the corresponding leaf node and “0” otherwise. The prediction of the entire ensemble in XGBoost is obtained by summing the predictions of all the decision trees and adding a bias term

F(x) = ∑ f_i(x) + b(14)

where f_i(x) is the prediction of the i-th decision tree, and b is the global bias.

▪ The gradient and Hessian of the objective function with respect to the predicted values in XGBoost are given by

g_i = ∂L(y_i, F(x_i))/∂F(x_i)(15)

h_i = ∂^2^L(y_i, F(x_i))/∂F(x_i)^2^ (16)

where y_i is the actual value of the i-th observation.

▪ The objective function of the H2OXGBoost algorithm is similar to that of XGBoost, but it includes additional terms for distributed computing

obj = (1/n) ∑L(y_i, F(x_i)) + λΩ(w) + γΦ(T)(17)

where “n” is the total number of observations, “λ” and “γ” are the regularization parameters, Ω(w) is the L norm of the weights in the decision trees, and Φ(T) is the complexity penalty that penalizes deeper trees. 

The following steps (the pseudocode) are used for MQTT attack detection when using the H2OXGBoost model:

1. Load the training data set
2. Preprocess the data by removing any missing values or outliers
3. Split the data set into training and testing sets
4. Initialize the H2OXGBoost algorithm with hyperparameters
5. Train the model on the training set using the train() function
6. Evaluate the model’s performance on the testing set using the evaluate() function
7. If the evaluation metrics are satisfactory, proceed to the next step. Otherwise, adjust the hyperparameters and retrain the model

The training and evaluation results of H2OXGBoost are documented in Table 3.

#### 4.4.3. H2O Deep Learning (DL) Algorithm 

H2O’s deep learning algorithm uses artificial neural networks with multiple hidden layers, known as deep neural networks, that are trained using stochastic gradient descent with backpropagation [41]. This algorithm can automatically perform feature engineering and includes features such as L1/L2 regularization, early stopping, and adaptive learning rates [42]. Unlike other algorithms, DL algorithms can use unlabeled data, which makes them suitable for several domains, including image classification [43], speech recognition, and natural language processing [44].

H2O’s DL algorithm for detecting MQTT attacks:

▪ Feedforward calculation:

h_i = f (W_i ∗ h_i−1 + b_i)(18)

where “h_i” is the output of layer i, “W_i” is the weight matrix of layer i, “b_i” is the bias vector of layer i, and “f” is the activation function.

▪ Error calculation:

E = (1/2) ∗ (y − y_predicted)^2^ (19)

where “E” is the mean squared error between the actual output “y” and predicted output “y_predicted”.

▪ Backpropagation:

delta_i = f′(z_i) ∗ ((w_i+1)^T ∗ delta_i+1)(20)

where “delta_i” is the error term for layer i, “w_i+1” is the weight matrix for layer “i+1”, “delta_i+1” is the error term for layer “i+1”, “f′” is the derivative of the activation function, and “z_i” is the weighted sum for layer i.

▪ Gradient descent:

W_i = W_i − a ∗ delta_w_i(21)

b_i = b_i − a ∗ delta_b_i(22)

where “W_i” is the weight matrix of layer i, “b_i” is the bias vector of layer i, a is the learning rate, and “delta_w_i” and “delta_b_i” are the gradients of the weights and biases, respectively.

▪ Dropout:

h′ = h ∗ r (23)

where “h” is the output of a layer in the network, “r” is a binary mask generated from a Bernoulli distribution with probability “p”, and “h′” is the output after applying dropout.

The following steps (the pseudocode) are used for MQTT attack detection when using H2O’s DL algorithm with a validation:

1. Load the MQTT traffic data into a data set.
2. Preprocess the MQTT traffic data by converting categorical variables to numerical forms and splitting it into training, validation, and testing sets.
3. Train a deep learning model on the training set using H2O’s DL algorithm. Specify the appropriate input features and output variables and choose appropriate hyperparameters (such as the number of layers, neurons, etc.). Then fit the model to the training data.
4. Use the trained model to predict the attack type of the validation set. Preprocess the validation data in the same way as the training set. Apply the trained model to the validation data to predict the attack type.
5. Analyze the predicted attack types to detect potential MQTT attacks. Flag MQTT traffic corresponding to a predicted malicious or suspicious attack type as potentially problematic.
6. If the performance on the validation set is satisfactory, use the trained model to predict the attack type of new MQTT traffic data. Preprocess the new data in the same way as the training/validation sets. Apply the trained model to the new data to predict the attack type.

The DL architecture applied for MQTT attack detection is presented in Table 4. It contains layers and the activation function within layers.

On the other hand, Table 5 presents a comprehensive performance analysis covering training and evaluation when utilizing H2O’s DL algorithm model for MQTT attack detection.

### 4.5. Discussion of Results

This work proposed a novel framework that addresses the challenges associated with brokers and the MQTT protocol. It achieves this by effectively analyzing and processing network traffic, employing an intelligent model to detect potential attacks that pose a threat to the protocol and broker operations. To ensure real-time activity and robust coordination between the broker and potential attack threats, DSBroker has been built and implemented. DSBroker leverages distributed analytics and intelligent detection capabilities to enhance the resilience of the broker against attacks. A key advantage of the proposed framework is the incorporation of H2O’s distributed and in-memory processing capabilities. This sets H2O at an advantage over the traditional machine learning approaches. By utilizing H2O’s cluster methodology, the proposed framework, DSBroker, is capable of processing traffic data in real time across a cluster of nodes. This distributed processing capability significantly enhances the efficiency of the framework. The proposed framework is implemented based on an environment that has the following details: H2O_cluster_version is 3.40.0.4, H2O_cluster_memory is 7.461 Gb, localhost, and Python_version is 3.10.10. A DSBroker-based H2O algorithm like H2OXGBoost for detection and coordination between IoT devices and sensors has the advantage of processing through a cluster of nodes, which enables the framework to run in real time. To provide further insight, the distributed processing in H2O is achieved through its cluster methodology, which involves the formation of an H2O cluster—a network of interconnected computing nodes/machines working collaboratively to perform distributed machine learning tasks. The process is conceptually described as distributed processing with an H2O cluster = Node_1_ + Node_2_ + … + Node_N_. Each individual node within the cluster represents a computing unit. Contrasted with a nondistributed approach, the elapsed time (ET_trad_) can be expressed as

ET_trad_ = D/S_trad_ (24)

where “S_trad_” denotes the speed of processing in a traditional, nonparallelized setting and “D” represents the size of the input data. 

The key comparison lies in the impact of parallelization within the H2O approach. Consequently, the elapsed time (ET) in H2O can be significantly lower than the traditional approaches (ET_trad_), owing to the higher speed of processing achieved through distributed processing (parallelization).

As described in Section 4.3, to evaluate the performance of different H2O models several built-in metrics are used. These metrics are mean squared error (MSE), root-mean-square error (RMSE), mean per class error (MCE), and log loss. These metrics are used to compare different models and select the best approach for the appropriate application. The following observations are derived from the data presented in Table 1, Table 2, Table 3, Table 4 and Table 5:

▪ The architectural details, training outcomes, and evaluation results of the DRF models are summarized in Table 1. The findings show a close resemblance between the results of the DRF model and other H2O methods.
▪ Table 2 provides a summary of the system performance observed when employing DGBM models for MQTT attack detection. The results indicate that model 2 outperforms model 1 in terms of accuracy, with slightly lower values of MSE, RMSE, log loss, and mean per class error compared to model 1.
▪ The training and evaluation results of H2OXGBoost are documented in Table 3. This table reveals that when H2OXGBoost is used for MQTT attack detection, it outperforms H2OGLM, H2OGBM, and H2ODL algorithms in terms of accuracy.
▪ Table 5 presents a comprehensive performance analysis covering training and evaluation when utilizing H2O’s DL algorithm model for MQTT attack detection.
▪ By examining Table 1, Table 2, Table 3 and Table 5, it becomes evident that the H2OXGBoost algorithm is the most accurate model compared to other H2O models for detecting MQTT attacks. Therefore, it is recommended to employ the H2OXGBoost model to enhance security in distributed and scalable environments, such as IoT networks, for protection against MQTT attacks.

To assess the effectiveness of the proposed model, a comparison is made with two recent models, considering the detection algorithm used, as well as the scalability and approach to distributed processing. Table 6 illustrates that the proposed model demonstrates superior scalability compared to the other models, excelling in distributed processing capabilities.

## 5. Conclusions

This work introduces a novel approach that leverages the distributed processing ML algorithm H2O to enhance network security against MQTT attacks. The proposed approach aims to achieve real-time detection and mitigation of potential security threats. The findings highlight the significance of employing the H2O distributed processing algorithm in strengthening network security and resilience against cyberattacks. Given the increasing reliance on IoT devices and the growing sophistication of cyber threats, it is crucial for the proposed model to continue exploring innovative solutions for mitigating these risks. The H2OXGBoost algorithm performs the best among the H2O models in terms of accuracy for detecting MQTT attacks. It outperforms other algorithms such as DRF, DGBM, H2OGLM, H2OGBM, and H2ODL. The evaluation metrics, including MSE, RMSE, log loss, and mean per class error, consistently show better results for the H2OXGBoost model. Therefore, it is recommended to use the H2OXGBoost model to enhance security in distributed and scalable environments, particularly in IoT networks, to protect against MQTT attacks. The authors believe that the proposed model holds substantial potential for practical applications, particularly in industries where data privacy and security are paramount. Moving forward, additional research and development efforts are necessary to refine and enhance the proposed model. Nevertheless, the authors are confident that this approach represents a promising direction in the ongoing endeavor to protect networks from MQTT attacks and other cyber threats. This paper aims to make a valuable contribution to this important research area and to inspire further investigations at the intersection of ML, network security, and distributed systems. Future studies in this area could look into ways to improve the current technique and meet new difficulties in the dynamic landscape of network security and IoT environments.

## Figures and Tables

**Table 1:** Performance analysis of the proposed model when using different DRF models for MQTT attacks.

DRF Models	Model Details		Performance Results		
**Model 1** Number of trees = 50	number_of_trees	50.0	**Metrics**	**Training**	**Validation**
	number_of_internal_trees	300.0	MSE	0.141	0.142
	model_size_in_bytes	111,129.0	RMSE	0.376	0.376
	min_depth	2.0	Log loss	0.404	0.403
	max_depth	20.0	Mean per class error	0.416	0.416
	mean_depth	9.23			
	min_leaves	1.0			
	max_leaves	435.0			
	mean_leaves	24.67			
**Model 2** Number of trees = 100	number_of_trees	100.0	**Metrics**	**Training**	**Validation**
	number_of_internal_trees	600.0	MSE	0.142	0.142
	model_size_in_bytes	222,104.0	RMSE	0.376	0.376
	min_depth	4.0	Log loss	0.401	0.407
	max_depth	20.0	Mean per class error	0.412	0.4208
	mean_depth	8.985			
	min_leaves	1.0			
	max_leaves	641.0			
	mean_leaves	24.66			
**Model 3** Number of trees = 50 and using k-fold cross-validation	number_of_trees	50.0	**Metrics**	**Training**	**Validation**
	number_of_internal_trees	300.0	MSE	0.142	0.140
	model_size_in_bytes	111,115.0	RMSE	0.377	0.375
	min_depth	2.0	Log loss	0.403	0.401
	max_depth	20.0	Mean per class error	0.416	0.416
	mean_depth	9.23			
	min_leaves	1.0			
	max_leaves	435.0			
	mean_leaves	24.6766			

**Table 2:** Performance analysis of the proposed model when using different DGBM models for MQTT attacks.

DGBM Models	Model Details		Results		
**Model 1** Number of trees = 50	number_of_trees	50.0	**Metrics**	**Training**	**Validation**
	number_of_internal_trees	300.0	MSE	0.126	0.126
	model_size_in_bytes	77.935.0	RMSE	0.355	0.356
	min_depth	1.0	Log loss	0.376	0.378
	max_depth	5.0	Mean per class error	0.401	0.403
	mean_depth	4.846667			
	min_leaves	1.0			
	max_leaves	29.0			
	mean_leaves	16.0433			
**Model 2** Number of trees = 100	number_of_trees	114.0	**Metrics**	**Training**	**Validation**
	number_of_internal_trees	684.0	MSE	0.125	0.126
	model_size_in_bytes	112,943.0	RMSE	0.354	0.355
	min_depth	0.0	Log loss	0.374	0.376
	max_depth	5.0	Mean per class error	0.394	0.397
	mean_depth	2.6140351			
	min_leaves	1.0			
	max_leaves	29.0			
	mean_leaves	8.387427			

**Table 3:** The performance analysis (training and evaluation) when using the H2OXGBoost model for MQTT attack detection and classification assuming that the trained model contains 100.0 number_of_trees.

Metrics	Training	Validation
**MSE**	0.100	0.101
**RMSE**	0.316	0.318
**Log loss**	0.367	0.371
**Mean per class error**	0.255	0.253

**Table 4:** Deep learning algorithm architecture parameters.

Layer	Units	Type	Dropout	l1	l2	Mean_Rate	Rate_rms	Momentum	Mean_Weight	Weight_rms	Mean_Bias	Bias_rms
1	43	Input	0.0									
2	200	Rectifier	0.0	0.0	0.0	0.316	0.433	0.0	0.016	0.130	0.319	0.108
3	200	Rectifier	0.0	0.0	0.0	0.441	0.425	0.0	−0.014	0.152	−0.156	0.250
4	6	Softmax		0.0	0.0	0.445	0.459	0.0	−0.569	0.882	−9.182	0.801

**Table 5:** Performance analysis (training and evaluation) when using H2O’s deep learning algorithm model for MQTT attack detection.

Metrics	Training	Validation
**MSE**	0.364	0.140
**RMSE**	0.603	0.375
**Log loss**	1.38	0.409
**Mean per class error**	0.430	0.426

**Table 6:** A gap analysis of DSBroker and other models.

Models	Detection Algorithm	Supportively the Scalability	Distributed Processing Manner
**ARTEMIS [45]**	SVM and k-means	X	X
**MQTT Attack Detection [46]**	SVM, k-means, random forest	X	X
**DSBroker**	H2OXGBoost and other algorithms	√	√

## Data Availability

Data are contained within the article.
